# An evolving perspective about the origins of childhood undernutrition and nutritional interventions that includes the gut microbiome

**DOI:** 10.1111/nyas.12487

**Published:** 2014-08-12

**Authors:** Tahmeed Ahmed, David Auble, James A Berkley, Robert Black, Philip P Ahern, Muttaquina Hossain, Andrea Hsieh, Santhia Ireen, Mandana Arabi, Jeffrey I Gordon

**Affiliations:** 1 Centre for Nutrition and Food Security, International Centre for Diarrhoeal Disease ResearchDhaka, Bangladesh; 2 Department of Biochemistry and Molecular Genetics, University of VirginiaCharlottesville, Virginia; 3 Clinical Sciences, KEMRI/Wellcome Trust Research ProgrammeKilifi, Kenya; 4 Department of International Health, Bloomberg School of Public Health, John Hopkins UniversityBaltimore, Maryland; 5 Center for Genome Sciences and Systems Biology, Washington University School of MedicineSt. Louis, Missouri; 6 The Sackler Institute for Nutrition Science, New York Academy of SciencesNew York, New York

**Keywords:** undernutrition, stunting, gut microbiota, nutrient–immune system interactions, gut barrier function, neurodevelopment and brain metabolism, environmental enteropathy, epigenetics, gnotobiotic mice

## Abstract

The Sackler Institute for Nutrition Science and the World Health Organization (WHO) have worked together to formulate a research agenda for nutrition science. Undernutrition of children has profound effects on health, development, and achievement of full human capacity. Undernutrition is not simply caused by a lack of food, but results from a complex interplay of intra- and intergenerational factors. Representative preclinical models and comprehensive well-controlled longitudinal clinical studies are needed to further understand the contributions and the interrelationships among these factors and to develop interventions that are effective and durable. This paper summarizes work on mechanisms underlying the varied manifestations of childhood undernutrition and discusses current gaps in knowledge and challenges to our understanding of undernutrition and infection/immunity throughout the human life cycle, focusing on early childhood growth. It proposes a series of basic and clinical studies to address this global health challenge.

## Context

Suboptimal growth is typically assessed by anthropometric measures that reflect stunting (height-for-age Z-score (HAZ)), underweight (weight-for-age Z-score (WAZ)) and/or wasting (weight-for-height Z-score (WHZ)). In 2011, estimates were that in low- and middle-income countries (LMICs), 165 million children under 5 years of age were stunted, 101 million were underweight, and 52 million were moderately or severely wasted.^1^

Stunting (low HAZ) is the most common manifestation of undernutrition. The periods between pregnancy and the first 2 years of life (the first 1000 days) are critical periods when intrauterine growth retardation and stunting develop.^2^ Some have claimed that up to one-third of stunting in early childhood is due to poor fetal growth.^3^ Following the first 1000 days, stunting generally does not worsen but is commonly irreversible, leading to adults with shorter stature.^4^

Women with a low body mass index (BMI) or short stature, especially if they conceive at a young age, have an increased rate of complications during pregnancy and delivery, leading to increased maternal morbidity and mortality as well as adverse perinatal outcomes.^5^ Women with low BMI or stunting have a substantially increased risk of having a baby born small for gestational age (SGA).^6^

**Figure 1:**
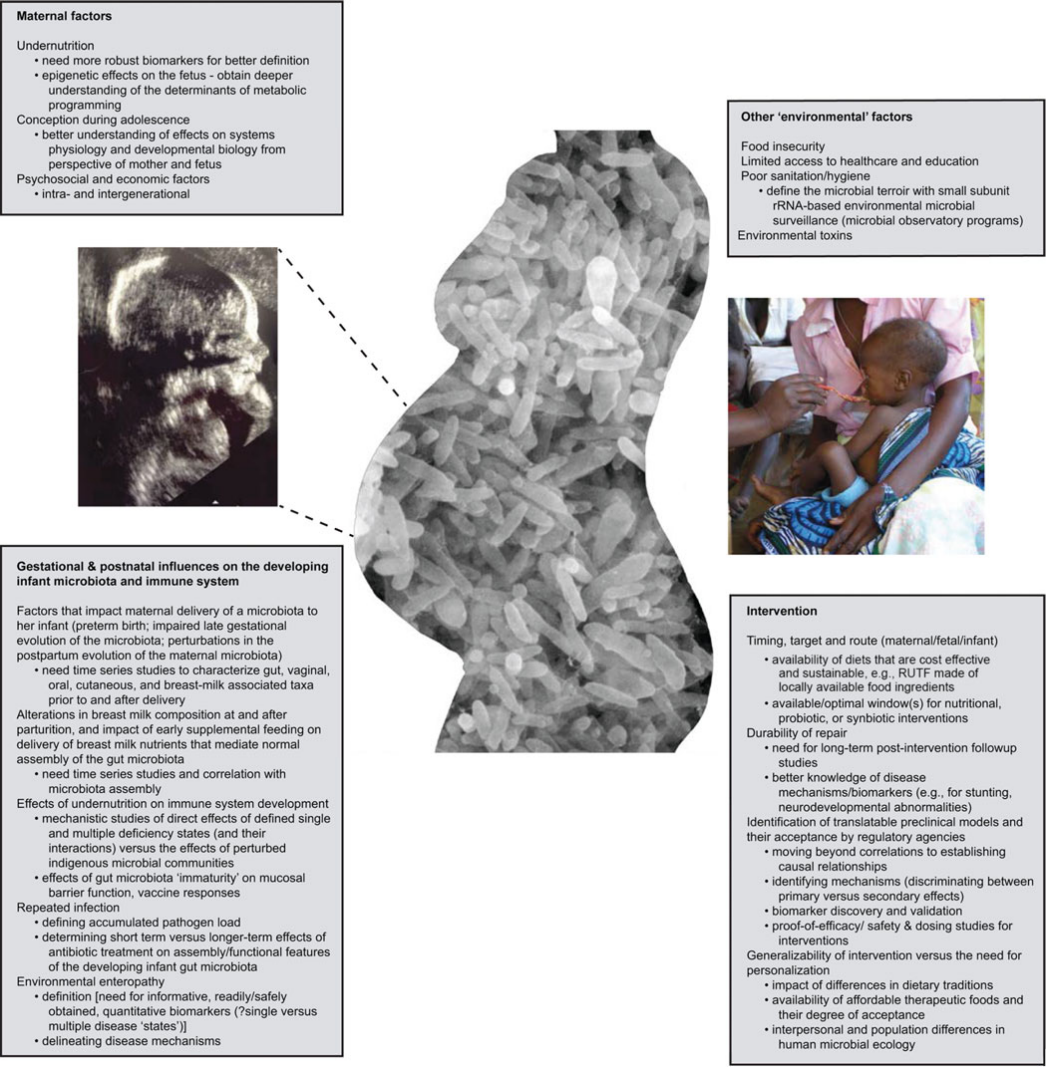
The determinants of stunting operate at multiple levels of causation.

The determinants of stunting operate at multiple levels of causation, ranging from the most distal socioeconomic and political variables to the most proximal, including the quantity and quality of food and its biotransformation by the gut microbiota, host infections, immune dysfunction, and associated perturbations in systems physiology (Fig.1). The quality of infant care affects both stunting and child development. The large socioeconomic inequalities underlying the prevalence of stunting in LMICs highlight the importance of the distal determinants. Epidemiological studies suggest that stunting in childhood is ascribable to suboptimal breastfeeding and complementary feeding practices, to micronutrient deficiencies, and to high rates of infectious diseases.^7^,^8^ A variety of contextual and environmental factors, including lack of maternal education and empowerment, poor hygiene and sanitation, and water and food contamination, are also important determinants.

Height is a heritable trait. Genome-wide association studies (GWAS) have identified ∼200 loci that have a significant association with height.^9^,^10^ Genes in these loci are involved in known biological processes that in many instances can be attributed to growth. Nonetheless, these loci explain only a small fraction (10%) of the observed variance in human height. Thus, not surprisingly, the genetic contributions to stunting are largely ill-defined, although the biological pathways associated with the identified loci can serve to help direct preclinical and clinical studies designed to assess the effects of various known or hypothesized environmental factors on the pathogenesis of stunting in at-risk populations. The role of environmental factors has been underscored by epidemiologic studies that have shown a reduction in the prevalence of stunting when members of a population where stunting is common migrate to different areas where socioeconomic status is improved.^11^

Reduced growth occurs in mice with a neuron-specific enolase–human interleukin-6 transgene (*NSE/hIl6*) compared to their nontransgenic littermates.^12^ *NSE/hIl6* mice produce very high levels of circulating IL-6 beginning at birth. Their growth defect is partially rescued with a monoclonal antibody to the murine IL-6 receptor. IL-6 treatment of nontransgenic littermates led to a significant decrease in IGF-1 levels, indicating that IL-6–mediated decreases in IGF-1 production is a pathway through which an inflammatory cascade can affect linear growth.^12^ The roles of IL-6 and other immunoinflammatory mediators as well as components of the growth hormone-IGF axis are areas that are being actively explored. For example, ghrelin, the endogenous ligand for the growth hormone secretagogue receptor GHSR type 1a, is produced in gastric enteroendocrine cells. While this orexigenic polypeptide induces secretion of growth hormone, its role in normal growth and stunting is unclear.^13^

Children with suboptimal growth exhibit increased incidence, duration, severity of infectious diseases, and risk of death from these infections.^14^,^15^ This risk increases monotonically as Z-scores decline (become more negative). Thus, undernutrition can be considered an underlying cause of death in a synergistic relationship with infections.^7^ Studies have consistently found that diarrhea is the most important infectious determinant of stunting. In a pooled analysis of nine community-based studies performed in low-income countries with daily household information on diarrhea and longitudinal anthropometry, the odds of stunting at 24 months of age increased multiplicatively with each diarrhea episode or day of diarrhea. The proportion of stunting attributed to five prior episodes of diarrhea was 25% (95% CI 8–38%).^16^

Stunting predicts poorer cognitive and educational outcomes later in childhood. At birth, the human brain represents approximately 12% of body weight (a value six times greater than in the adult). The brain's energy demands are great in childhood. The metabolic rate of the newborn brain is lower than in the adult^17^,^18^ (presumably lessening nutritional demands given the increased brain-to-body-weight ratio). By age 2, blood flow and metabolism in the brain reach adult levels, while at the end of the first decade of life glucose metabolism and blood flow are almost twice adult levels (at a time that the child's brain-to-body-weight ratio remains several fold higher than in adults). Blood flow and metabolism finally achieve adult levels during the third decade. Postnatal changes in brain metabolism during the first 2 decades coincide with synaptic proliferation and subsequent pruning.

Key questions include how undernutrition in utero and during the postnatal period affects regional brain metabolism and neural development; whether, how, and for how long nutritional interventions directed at children with stunting and/or wasting affect these processes; and what roles the energy and metabolic output of the gut microbiota play in determining whether the energy and metabolic needs of the developing postnatal brain are met adequately. Functional magnetic resonance imaging (fMRI) and positron emission tomography (PET) provide ways of addressing these questions with quantitative imaging biomarkers.

Micronutrient deficiencies have adverse effects on myriad aspects of physiology including neural and immune functions. For example, subclinical vitamin A deficiency is prevalent, affecting an estimated one-third of children and 15% of pregnant women,^19^ and increases the risk of death from diarrhea (and measles). An estimated 17% of the world's population consumes diets that lack adequate amounts of zinc to satisfy metabolic needs.^20^ Zinc deficiency impairs immune function and increases the incidence of mortality owing to pneumonia and diarrhea. Iron-deficiency anemia is common in children and is associated with poorer cognitive and motor development. Iron supplementation in iron-deficient schoolchildren generally benefits cognition, although trials in children under 3 years of age have been inconsistent in finding benefits.^21^–^23^ Mild forms of iodine deficiency are extremely prevalent globally,^24^ despite the use of iodized salt, which is said to reach over two-thirds of the world's population. However, the biological consequences of mild deficiency are not well understood.

## Environmental enteropathy and stunting

Environmental enteropathy (EE) appears to have an important effect on stunting.^25^ This enigmatic disorder is characterized by villus blunting, reduced intestinal epithelial surface area and absorptive capacity, altered gut mucosal barrier integrity, and immunoinflammatory changes. EE occurs in young children living in unsanitary settings.^26^ These children experience high rates of symptomatic and asymptomatic enteric infections, but the interrelationships among these infections, the structural and functional configurations of their gut microbiota, the maturation and expressed properties of the innate and adaptive arms of their immune systems, their various environmental exposures, and the EE pathogenesis remain unclear.

Human communities where hygiene and sanitation are poor have widespread environmental fecal contamination. Globally, 86% of fecal pollution in the environment comes from livestock, compared to only 14% from humans.^27^ Children living in highly contaminated environments are frequently exposed to animal excreta via water, air, uncovered food, and more frequently via their own contaminated fingers. Moreover, there is higher pathogen load found in both human and animal populations living in close proximity to each other.^28^ However, members of communities exposed to poor hygienic conditions are not equally affected by EE,^25^,^26^ indicating that additional underlying causative factors conspire to produce disease. Nonetheless, a commonly held view of EE pathogenesis is that constant exposure to water and food contaminated with fecal bacteria persistently activates the immune system. Luminal bacteria stimulate pattern-recognition receptors (PRRs, including Toll-like receptors (TLRs)) and inflammasomes expressed by various cell types of the innate system as well as by epithelial cells. PRR-mediated recognition of microbe-associated molecular patterns (MAMPs), subsequent activation of PRR-associated signaling pathways, functional breakdown of epithelial tight junctions, increased paracellular leakage (in part through activation of intraepithelial lymphocytes (IELs)), the resulting T cell activation, and production of proinflammatory cytokines with subsequent recruitment of additional immune effector cells together produce a self-reinforcing pathogenic cascade that ultimately yields a remodeled intestinal architecture with lymphocytic infiltration in the lamina propria, villus atrophy, and worsening nutritional deficiencies.^29^ Nutritional deficiencies, in turn, contribute to impaired rates of epithelial renewal.

In this commonly held view of EE, growth falters when these changes coincide with marginal dietary intake and the high nutrient requirements for sustaining growth in the first 2 years of life.^30^ Changes in intestinal permeability may not be reversed with appropriate supplemental feeding, while alterations in gut architecture and barrier function limit responses to nutritional interventions.^31^ To date, nutritional interventions targeting children with EE have shown little effect on linear growth.^29^,^32^

Numerous research gaps related to EE have been highlighted in many publications.^25^ The role of the human gut microbiota in the development of EE needs to be explored for a variety of reasons. The microbiota influences the bioavailability and metabolism of macro- and micronutrients, the development of the immune system, gut mucosal barrier function, proliferation of epithelial cell progenitors in the crypts of Lieberkuhn, differentiation of the intestine's four epithelial cell lineages and their expressed functions, and susceptibility to invasion with enteropathogens. While alterations in mucosal barrier function are important determinants of pathogen exposure, chronic immune stimulation has been implicated in what has been termed *immune inefficiency*.^33^,^34^ Additional tools are needed to assess various facets of barrier integrity *in vivo*. Currently, no definitive noninvasive diagnostic tests for EE exist, and assessing therapeutic interventions for EE remains a great challenge. Representative preclinical models that emulate the structural and functional features of EE are needed to develop and test hypotheses about the underpinnings of this disease or diseases and its (their) manifestations, including the relationship among EE, nutritional status, and growth. Such models may help identify mechanism-based or surrogate biomarkers for disease classification, patient stratification, and ways of monitoring responses to therapeutic interventions, including the degree to which such responses are sustained.

## Undernutrition and the immune system

Infections typically reduce the intake of nutrients and their bioavailability, increase nutrient and energy expenditure, and divert nutrients away from growth.^8^,^25^ Subsequent catch-up growth depends on the extent of prior nutritional deficiencies, nutritional intake, and the duration of the period before the next infection. While epidemiological data supporting the associations described above are strong, there is a lack of clear understanding of the mechanisms involved.^8^ More detailed physiological, metabolic, immunologic, and genomic studies are needed to understand the protein, energy, and micronutrient requirements of various populations of children over a range of ages, during and after infection.

Attempts to characterize immunodeficiency attributable to undernutrition have resulted in unclear or conflicting findings, in part because of varied clinical study designs, the varied nature of concurrent infections, and the varied types and differential effects of macro- and micronutrient deficiencies. Nonetheless, recent data from human studies and from animal models have increased our understanding of the molecular mechanisms involved in immune functions that are dependent on various nutrients. Three notable examples involve tryptophan, vitamin A, and the short-chain fatty acid (SCFA) products of gut microbial fermentation of dietary glycans.

Tryptophan is an essential amino acid and thus is required for many cellular processes. A lack of tryptophan is detrimental to many infectious agents, in addition to the hosts they are attempting to infect. Indeed, the host has evolved the capacity to catabolize tryptophan and deplete it from the local milieu through the activity of a number of enzymes. Two such enzymes are well represented in immune cells, indoleamine 2,3-dioxygenase (IDO)^35^ and tryptophan hydroxylase 1 (TPH1).^36^ Tryptophan depletion has a number of effects. Important among these is the restriction of pathogen growth (group B streptococci, *Mycobacterium avium*, cytomegalovirus)^37^–^39^ and the inhibition of the inflammatory function and proliferation of T cells. This latter aspect is the best studied of the two effects, with tryptophan depletion thought to be an active mechanism used to generate tolerogenic immune responses. IDO is expressed by antigen-presenting cells (APCs) (dendritic cells (DCs), plasmacytoid DCs, and macrophages)^35^,^40^ and can be induced by inflammatory mediators, such as IFN-γ,^40^,^41^ the combinatorial activity of IFN-γ, TNF-α, and IL-1β,^42^ or ligation of CD80/CD86 with CTLA4-Ig.^43^ IDO^+^ APCs can limit T cell proliferation and induce cell cycle arrest by limiting the tryptophan that is critical for T cell function. The lack of available tryptophan is sensed directly by T cells themselves through the amino acid–starvation response (CD8^+^ T cells deficient in GCN2 stress-response kinase are refractory to suppression by tryptophan depletion).^44^

A striking aspect of IDO function is its requirement to maintain tolerance to the allogeneic fetus (i.e., to allow mothers to carry to term offspring that express antigens (from the male) that are foreign).^45^ IDO activity has also been implicated in limiting the severity of inflammation in mouse models of inflammatory bowel disease (trinitrobenezene sulfonic acid (TNBS) colitis)^46^ and multiple sclerosis (experimental autoimmune encephalitis (EAE)).^47^ IDO is further required for acquisition of tolerance to neoantigens such as grafts.^43^,^48^ Moreover, the beneficial effects of CTLA4-Ig, a fusion protein that promotes immune tolerance through blockade of important costimulatory pathways, are largely dependent on IDO function in the host to which it is administered.^43^,^48^ Interestingly, metabolites generated by IDO-mediated catabolism of tryptophan have also been posited to possess immune-suppressive function,^49^–^51^ including the ability to inhibit autoimmune inflammation in mice.^51^ Less is known about the role of tryptophan hydroxylase 1 (TPH1) in immune regulation, but a recent report showed that it was expressed by mast cells, that it could deplete tryptophan, and that its expression plays a role in (1) maintenance of tolerance to grafts, (2) limiting the severity of EAE, and (3) promotion of tumor growth.^36^

Gut bacteria are equipped with the capacity not only to synthesize tryptophan, but to catabolize it to kynurenine. Bacteria with the capacity to generate kynurenine were reported to be enriched in the guts of HIV^+^ patients compared to HIV^−^ controls. Moreover, a positive correlation was observed between plasma kynurenine/tryptophan ratios and IDO expression in untreated HIV^+^ patients compared to HIV^+^ patients undergoing highly active antiretroviral therapy (HAART), suggesting higher activity of this pathway among the untreated patients.^52^

Together, these studies reveal a profound role for tryptophan depletion in the active induction of T cell tolerance and demonstrate the central role played by tryptophan in the control of immune responses.

The predominant effects of vitamin A on the immune system are thought to be mediated by retinoic acid (RA). RA is generated from dietary vitamin A through a number of steps, the final being conversion of retinal to RA by retinal dehydrogenase.^53^ Although various cell types can perform this transformation, APCs, specifically those at tissue sites like the intestine and skin, are specialized in the provision of RA for modulation of immune responses. APCs express high levels of the enzyme retinal dehydrogenase and can thus modify immune responses through provision of RA^54^–^56^ to cells with which they are interacting.

RA has been strongly linked to modulation of intestinal immune responses. RA promotes the upregulation of the gut homing receptors CCR9 and α_4_β_7_ integrin on B cells and aids in the generation of IgA^+^ B cells. RA also modulates the function and phenotype of both CD4^+^ and CD8^+^ T cells, promoting expression of CCR9 and α_4_β_7_ and enhancing gut homing function.^54^,^57^,^58^ In addition, RA helps shape helper T cell differentiation in several ways. First, RA can synergize with the activity of TGF-β to promote development of induced FoxP3^+^ T_reg_ cells,^55^,^59^–^61^ although it should be noted that neither the frequency nor number of T_reg_ cells are reduced in vitamin A–deficient mice.^62^ Second, RA is required to mount protective immune responses to infection by organisms such as *Toxoplasma gondii*.^62^ Third, vitamin A–deficient mice lack T_H_17 cells^63^,^64^ and display impaired T_H_1 and T_H_17 responses to *T. gondii* infection and oral administration of *Escherichia coli* heat-labile enterotoxin.^62^ Vitamin A is also required for conditioning of DCs and promoting the stereotypical phenotypic features of intestinal CD103^+^ DCs,^58^ highlighting the fact that multiple cell types are modulated by vitamin A and underscoring the important role it plays in immunity.

Intriguingly, bile, which contains retinol, has been demonstrated to drive the expression of the gene encoding retinaldehyde dehydrogenase 2 (RALDH2; responsible for generating RA from retinaldehyde) in DCs, through a process that depends on RA signaling, and further conditions DCs to induce gut-homing potential (CCR9 expression) in CD8^+^ T cells *in vitro*.^58^ Given the profound effects of the gut microbiota on bile metabolism/composition, understanding the impact of the microbiota of undernourished versus healthy children on the pool of retinoids in bile could provide important mechanistic insights into the interrelationships between diet, the microbiota, and various aspects of immune function.

SCFAs have long been known to play an important role in host physiology. More recently, their potential for immunomodulation has come to be appreciated. Acetate aids in the resolution of dextran sodium sulfate (DSS)–induced intestinal inflammation through the G protein–coupled receptor GRP43^65^ and promotes epithelial barrier integrity, thereby reducing mortality in an *E. coli* 0157:H7 infection model in mice.^66^ Garrett and coworkers demonstrated that administration of SCFAs via drinking water promotes development of colonic T_reg_ cells.^67^ Although acetate, propionate, and butyrate could drive the response, acetate appeared to be the most potent of the three SCFAs tested. Moreover, T_reg_ cell induction by propionate required expression of GRP43. A cocktail of acetate, butyrate, and propionate enhanced T_reg_ cell suppressive activity in the T cell transfer colitis model in mice; the enhanced suppressive activity in response to propionate also required GPR43 expression in T_reg_ cells.^67^

Colonization with Clostridia recovered from the human intestinal microbiota is sufficient to increase T_reg_ cell accumulation in the mouse colon: this induction is associated with increases in SCFA levels and has been proposed to be linked to the ability of SCFAs to stimulate TGF-β expression from epithelial cells, as happens *in vitro*.^68^

Collectively, the above data suggest that microbial metabolites modulate immune function, highlighting the value in understanding what types of metabolites the microbiota produces as a function of diet, the age of the host, and growth phenotype/nutritional status.

## Pregnancy

Fetal growth and development are influenced by a complex interplay between maternal nutritional status and nutrient intake (before and during pregnancy); metabolic, endocrine, and immune functions; and placental biology. There are too few biomarkers available for defining maternal nutritional status before conception, at various stages during pregnancy, and following parturition. One obvious parameter to use in trial design and interpretation is the presence or absence of a given nutritional intervention. Macro- and micronutrient supplementation have modest reported effects on birth weight and preterm birth. For example, zinc supplementation in pregnancy results in some reduction in preterm birth, but has no effect on birth weight or neonatal survival. Vitamin D deficiency in pregnancy is associated with bacterial vaginosis,^69^ which itself is associated with preterm birth. Low cord blood vitamin D levels are a predictor of subsequent infant respiratory infections.^70^,^71^ However, data from clinical trials are too limited to draw conclusions about the efficacy and safety of vitamin D supplementation in pregnancy.^72^ Meta-analyses have highlighted the need to extend endpoints in intervention trials of multiple micronutrients in pregnancy.^30^ The timing of nutrient interventions during pregnancy or before conception is an important clinical study parameter to consider in any meta-analysis designed to evaluate the effects of an intervention or interventions and the observed effect size.

## Epigenetics

Work over the last several decades has shown that environmental influences, including nutrient insufficiency and exposure to toxic substances, can affect the health and behavior of individuals many years later, or even the health and behavior of offspring who never directly experienced the original environmental influence.^73^,^74^ Stable reprogramming of gene expression during a developmentally plastic interval is thought to predispose individuals to future disease if the future environment is different from the one in which the expression program was established.^75^ On a molecular level, there is strong evidence, particularly in mice, that changes in DNA methylation underlie this type of epigenetic “memory.” Studies in rodents have taken advantage of uniform genetic backgrounds and well-controlled environments to provide molecular support for the long-term epigenetic effects of folate, as well as the ubiquitous endocrine disruptors bisphenol A and vinclozolin, via DNA methylation.^76^,^77^ An emerging body of evidence suggests that a wide range of environmental substances can exert such effects, and investigators have begun to document their interplay, along with the epigenetic consequences of nutrient limitation, in humans.^77^

A notable example of how epigenetic studies may affect treatment in response to the dual insults of undernutrition and toxin exposure is the recent work demonstrating an effect of arsenic on DNA methylation. While the biological effects of arsenic are complex, the data suggest a model in which arsenic perturbs one-carbon metabolic flux, potentially explaining, at least in part, how arsenic exposure exacerbates the long-term health effects of undernutrition.^78^ It is plausible that analogous studies investigating epigenetic profiles resulting from other toxin exposures will offer insight into both prognosis and treatment.

Consistent with rodent studies, undernutrition and, conversely, micronutrient supplementation are associated with significant changes in locus-specific DNA methylation in humans.^79^ However, the epigenetic effects of undernutrition and toxin exposure are in general not well documented or understood in humans. To date, relatively few genome-wide studies have been conducted. There is very little known about the relative contributions of the paternal and maternal epigenomes to human transgenerational inheritance in different settings.^80^ For many toxic substances of widespread concern, including aflatoxin, lead, cadmium, and pesticides, and for many illicit drugs that are commonly abused, there are little or no data available assessing whether, and to what extent, exposure correlates with changes in DNA methylation. There is limited dose–response data even in rodent models, especially for doses corresponding to human exposure levels. As populations are often exposed to toxins in combination, there is a pressing need to understand the combinatorial epigenetic effects of toxins, particularly with regard to how they influence nutritionally vulnerable populations.

It is unlikely that the epigenetic memory of a prior environmental condition is recorded solely by changes in DNA methylation. Histone posttranslational modifications and RNA-based mechanisms also play critical roles in epigenetic control.^81^,^82^ The availability of acetyl-, phospho-, and methyl-donors are likely tied to nutritional status and to some degree the metabolic activities expressed by the microbiota (see below). Moreover, DNA methylation, histone modification and RNA-mediated regulatory processes are intimately interconnected at a molecular level, and may regulate one another or interact synergistically to reinforce an environmental effect. Among myriad histone modifications, lysine and arginine methylation are well known to influence gene expression globally during development,^83^ and it is likely that histone methylation is affected by nutritional status and toxin exposure, just as DNA methylation is affected.

The potential power of uncovering genome-wide epigenetic signatures of prior environmental exposure is that such data may ultimately be of predictive value, in that they may allow for the identification of individuals living in the same general environment with different long-term health prognoses. They may also point to currently unsuspected molecular targets for intervention.

Hope for progress is not only based on advances in DNA sequencing that have allowed the epigenome to be more comprehensively profiled at ever diminishing cost, but also on new methods. One such method couples multiplex bisulfite sequencing with laser capture microdissection techniques to define genome-wide DNA methylation patterns in tissue samples. Laser capture microdissection–reduced representation bisulfite sequencing (LCM-RRBS) can map DNA methylation across most CpG islands and gene promoters using DNA extracted from as a little as 2 mm^2^ of a microdissected fresh frozen tissue section or 20 mm^2^ of a formalin-fixed paraffin-embedded tissue section.^84^

## The role of the gut microbiota

The human gut is home to tens of trillions of microbes that belong to all three domains of life (Bacteria, Archaea, and Eukarya), and their viruses. Membership is dominated by components of bacteria.^85^–^88^ Assembly of this microbial community (collectively referred to as the *microbiota*) and its repertoire of microbial genes (*microbiome*) begins at birth. In healthy individuals, this microbial “organ” evolves toward an adult-like configuration during the first three years of life.^89^ Interpersonal differences in gut microbial community membership are greater than intrapersonal variations, with family members sharing a significantly greater degree of similarity than unrelated individuals living in different households.^85^,^89^,^90^

Comparative culture-independent (metagenomic) analyses of the fecal microbiota collected from infants, children, and adults living on three continents in three countries representing different cultural traditions have disclosed features of a maturational program where the proportional representation of genes encoding functions related to micro- and macronutrient biosynthesis and metabolism changes during postnatal development. Aspects of this program are shared across populations, while other features distinguish Western from non-Western gut communities.^89^

An increasing number of clinical and preclinical studies indicate that food is a dominant factor that shapes the configuration of the gut microbial community; that the community, in turn, plays a role in defining the energetic/nutritional value of food for the consumer; and that the metabolic output of diet-by-microbiota interactions has myriad effects on host biology.^91^–^97^ As noted in the preceding sections of this paper, epidemiologic studies have shown that food insecurity alone does not explain the incidence of malnutrition in various populations. Together, these observations suggest that the gut microbiota/microbiome provides essential functions needed for healthy postnatal growth and development. A testable hypothesis is that disturbances in the assembly of the gut microbiota/microbiome, including disturbances related to enteropathogen infection, affect the risk for undernutrition and that undernutrition, in turn, affects community microbiota functions involved in determining nutritional status, thus further worsening health status.^94^,^98^,^99^

A challenge in testing this hypothesis is that there may be several different gut community configurations (states) associated with moderate or severe forms of acute undernutrition among different individuals and even within a given individual over time. Moreover, these configurations may be differentially affected by a given therapeutic intervention (e.g., antibiotics and ready-to-use therapeutic foods (RUTFs)), by different types of interventions, and/or by the timing/duration of the interventions. In addition, features of the gut community that are reconfigured during treatment may not persist after withdrawal of treatment.

Given the substantial interpersonal differences that exist between the microbiota/microbiomes of unrelated individuals and the confounding effects of postnatal age and different environmental exposures on microbiota/microbiome configuration (e.g., microbes emanating from contact with the mother and other family members, interpersonal differences in the nutrient content of breast milk, or intrapersonal variations that occur as a function of time after parturition), twins discordant for undernutrition provide an attractive study design for addressing the questions posed above. In countries where the standard of care is to give both twins in a pair discordant for severe acute malnutrition (SAM) RUTF to minimize food sharing by the mother (thus decreasing the risk that the healthy co-twin will develop SAM), each sibling can serve as his/her own control (comparing his/her microbiota/microbiome just before, during, and after treatment). In addition, the healthy twin in the discordant pair provides a control matched in age and, to a large extent, diet and other environmental exposures. Finally, age-matched twin pairs in the population who are concordant for healthy status serve as yet another set of reference controls.

These points were illustrated by a recent study of >300 mono- and dizygotic twin pairs who lived in five rural villages in southern Malawi. Twins were followed until they reached 36 months of age.^94^ During this period, 50% remained healthy, while 43% became discordant and 7% manifested concordance for acute moderate or severe malnutrition. Remarkably, the rate of moderate acute malnutrition (defined as WHZ between −2 and −3) or SAM (WHZ less than −3 or, in the case of kwashiorkor, generalized edema) was not significantly different in monozygotic compared to dizygotic twin pairs. This finding emphasizes the importance of factors beyond human genotype in disease pathogenesis. Turning to these individuals’ microbial genes, sequencing fecal community DNA prepared from same-gender twin pairs discordant for kwashiorkor disclosed that this form of SAM was associated with a functionally immature microbiome (as defined by the proportional representation of microbial genes with assignable enzyme classification numbers or Kyoto Encyclopedia of Genes and Genomes (KEGG) orthology groups (KOs)). While administration of a peanut-based RUTF promoted functional maturation of the kwashiorkor microbiome, the effect was short-lived, ceasing once RUTF was withdrawn.^94^

To determine whether these changes in the microbiome were causally related to disease, fecal samples from each co-twin in several twin pairs discordant for kwashiorkor were transplanted into the intestines of separate groups of adult germ-free mice (human donor samples were obtained at the time of diagnosis of kwashiorkor, before treatment had been initiated). The resulting humanized gnotobiotic animals were fed a representative macro- and micronutrient Malawian diet for several weeks, followed by RUTF for several weeks, followed by a return to the Malawian diet, thereby simulating features of clinical practice.

Transplantation of the human microbiota was efficient, as judged by capture of bacterial species and microbial genes present in the donor's sample, and reproducible within a group of recipient mice within an experiment and across repeated experiments. Significant differences in the proportional representation of bacterial species were readily detected in recipients of kwashiorkor compared to healthy co-twin gut communities, while animals were consuming the Malawian diet (e.g., increased representation in the kwashiorkor microbiota of *Bilophila wadsworthia*, a sulfite-reducing organism that induces a proinflammatory T_H_1 response under certain dietary contexts in mice and is linked to inflammatory bowel diseases in humans). In addition, fecal samples from discordant twin pairs transmitted a discordant weight-loss phenotype to recipient mice. This phenotype was diet-dependent; gnotobiotic mice harboring a kwashiorkor microbiota and consuming a nutrient-deficient Malawian diet exhibited significantly more weight loss than mice with a kwashiorkor microbiota that were fed a control nutrient-sufficient diet, or mice with a healthy co-twin microbiota that were fed a Malawian diet.^94^

Peanut-based RUTF produced significant increases in the proportional representation of a number of potentially beneficial species in gnotobiotic mice with a kwashiorkor co-twin's microbiota, including members of Bifidobacteria (*Bifidobacterium longum*, *B. bifidum*), Lactobacillus (*Lactobacillus reuteri, L. gasseri*), and Faecalibacteria (*Faecalibacterium prausnitzii*). Nonetheless, these changes were transitory and did not persist after withdrawal of RUTF.^94^ The transplanted kwashiorkor gut community was also metabolically labile, changing its expressed metabolic features upon exposure to RUTF, but not in a sustained way when RUTF was withdrawn. The combination of a nutrient-deficient Malawian diet and a kwashiorkor microbiota produced a variety of metabolic derangements, including deficiencies in essential amino acid metabolism, sulfur metabolism, carbohydrate fermentation, and inhibition of the microbiota's and the host's TCA cycle.

While the gut microbiomes of additional individuals representing different populations of children with various forms of SAM, sampled at various time points during disease and recovery, need to be characterized using the approaches described above, these results have a number of implications.

They indicate that the combination of a nutrient-deficient diet and perturbed microbiota/microbiome plays a significant role in disease pathogenesis.

The results suggest that the abnormalities associated with kwashiorkor microbiomes are not fully repaired with short-term RUTF treatment and that more sustained nutritional support with existing or different RUTFs may be required to ameliorate or prevent long sequelae of SAM, including persistent stunting and neurodevelopmental abnormalities.

As noted above, the developing brain poses considerable nutrient requirements that are unlikely to be met in situations where there is undernutrition affecting a mother, her fetus, and/or her infant/child. Humanized gnotobiotic mice provide a way of exploring whether and how the gut microbiomes of malnourished mothers, infants, or children, combined with nutrient-deficient diets, may contribute to disorders in brain metabolism. As such, these mice could provide a preclinical rationale for and direct the design of clinical studies.

The high incidence of discordance for moderate acute malnutrition and SAM in monozygotic and dizygotic twins raises a number of mechanistic questions. Early perturbations in as-yet poorly understood factors that shape the trajectory of microbiota/microbiome development (e.g., enteropathogen infection, antibiotic treatment, breast milk nutrient content and feeding history, and facets of immune and nonimmune host responses) may alter the configuration of the gut community, define its degree of volatility versus stability (or, put another way, ecosystem resilience, preservation of ecosystem services, and their capacity to be restored), and shape its functional maturation in one but not the other co-twin.

An intriguing question is how the metabolic output of the microbiome may imprint itself on the host during this formative stage of microbial community development. The developmental program of gut microbiome maturation described above includes changes in the representation of genes involved in the biosynthesis of folate and cobalamin—two vitamins that serve as methyl donors. As such, perturbations in this maturational program could affect the degree of methylation of host DNA. A recent study showed that the level of methylation of the *Tlr4* gene is significantly lower in the colons of germ-free compared to conventionally raised mice, illustrating a mechanism by which the microbiota could affect facets of innate immunity.^100^ Twins discordant for SAM would provide a very attractive study paradigm to examine the impact of malnutrition on host DNA methylation.

In addition to these epigenetic effects, the epiproteome may also be a target of the microbiota. Microbial fermentation of dietary polysaccharides generates SCFAs, including acetate and butyrate: the former can contribute to cellular acetyl-CoA pools; the latter is an inhibitor of protein lysine deacetylases. A high-resolution quantitative proteomic study, conducted in (1) adult germ-free mice uniformly labeled with ^15^N (through consumption of ^15^N chow for two generations), and (2) ex-germ-free animals that had received a gut microbiota transplant (in adulthood) from conventionally raised mouse donors, revealed a mechanism by which gut microbial community metabotype can be impressed on the host. Multiple mitochondrial, nuclear, cytosolic, and secreted proteins underwent microbiota-associated increases in their levels of lysine ε-acetylation (including residues located at or near the active sites of enzymes involved in functions related to energy production, respiration, and primary metabolism) in both the gut and liver.^101^ Quantitative epiproteomic studies in gnotobiotic animals hold promise for enhancing our understanding of how diet-by-microbiota interactions affect host biology and for identifying new biomarkers of nutritional status.

The relative impact of micro- versus macronutrient deficiency on the gut microbiota needs to be explored further. Humanized gnotobiotic mice provide an opportunity to test hypotheses about whether specific dietary micronutrient deficiencies have differential effects on the human gut microbiota, whether and how these effects could impact the fitness and niche (profession) of community members, features of community metabolism and host biology, and whether and how these effects may be long lasting, particularly if they occur during the period of gut microbial community assembly and functional maturation, even if dietary micronutrient sufficiency is subsequently restored. In addition, it may be very informative to characterize the evolved responses of gut microbial strains that have adapted to long-term micronutrient deficiency in their host human populations

To achieve persistent correction of abnormalities present in the gut communities of infants and children with SAM (or moderate acute malnutrition), it may be necessary to treat affected individuals with microbial species that perform functions insufficiently represented in the microbial communities of these infants and children. There is scant evidence that existing fermented milk product–associated probiotics will provide durable repair of key microbiota functions. For example, the randomized placebo-controlled Pronut Malawi trial examined the effects of treating children with SAM with RUTF versus RUTF and a synbiotic consisting of a consortium of four lactic acid bacterial species (*Pediococcus pentosaceus*, *Leuconostoc mesenteroides*, *L. paracasei* ssp. paracasei, and *L. plantarum*) plus four fermentable fibers (oat bran, inulin, pectin, and resistant starch). No statistically significant improvements in growth, infections, or mortality were observed compared to RUTF alone, although there was a trend toward reduced mortality.^102^

The source of probiotics in the future will likely be the human gut communities of healthy individuals from the same populations or different populations where disease is manifest. Obtaining these next-generation probiotics will be helped by recent advances in generating and characterizing clonally arrayed, sequenced bacterial culture collections from the frozen fecal microbiota of individuals representing ages and physiological phenotypes of interest and by their introduction into and characterization in preclinical gnotobiotic models.^103^,^104^ Well-controlled clinical trials are needed to assess how factors such as (1) the timing of initiation, (2) the duration of administration, and (3) the route of administration (e.g., directly to the child, through the mother, or a combination of both) of existing or next-generation RUTFs and of probiotics effect repair of the microbiota and restore normal metabolic functions and healthy growth in a durable manner. Fortunately, gnotobiotic animal models fashioned using human microbiota and diets representative of the very population(s) targeted for disease treatment and prevention are a source of new microbial and metabolic biomarkers that could be used to judge the safety and efficacy of these envisioned microbiota-targeted therapeutic interventions.

Finally, when considering the microbiota and its role in undernutrition, it is important to point out that clinical trials testing the efficacy of oral vaccines against polio, rotavirus, and cholera have shown lower immunogenicity in individuals from developing countries when compared to individuals from the developed world.^105^,^106^ To maximize the protective effects that can be achieved with oral vaccines in developing countries, it is crucial to understand why immune response and efficacy tend to be lower in such populations than in populations in industrialized countries. This variation in vaccine response may reflect a number of factors including socioeconomic conditions, nutritional status, and host genetics.^107^ Another hypothesis is that the structural and functional configuration (maturational state) of the intestinal microbiota plays a role in defining vaccine efficacy. This area of research is still in its infancy, including studies of how the microbiota and immune systems codevelop in healthy versus undernourished children (with and without EE).

## Synopsis

Undernutrition is not simply the result of a lack of food. A more holistic picture is emerging, in which environmental factors, relationships with pathogenic and nonpathogenic microbes, inflammation and immune programming, and long-term intra- and intergenerational effects, as well as specific nutritional deficiencies, lack of exclusive breastfeeding, and suboptimal complimentary foods, are involved in the genesis of childhood undernutrition. In turn, many of these factors are influenced by nutritional status, and each interacts with the others, creating a complex system. There is increasing recognition that undernutrition and its associated factors have long-term consequences for health and productive capacity, and for future generations.

This field is inherently interdisciplinary, and innovative programs need to be developed to attract students and established scientists with different disciplinary interests in the social and biological sciences and engineering (e.g., the need to develop new strategies for safe and sustainable improvements in sanitation as illustrated by the Reinvent the Toilet Challenge from the Bill & Melinda Gates Foundation) so that they are engaged in this global health problem.

New experimental and computational tools that support systems-wide characterization of physiology, metabolism, and immunity in preclinical models and in humans offer exciting possibilities for understanding the mechanisms that produce disease, for developing new biomarkers to define disease states, and for identifying next-generation therapeutics. Clinical trials of nutritional interventions offer opportunities to develop and evaluate the short- and long-term consequences of undernutrition, identify disease mechanisms, and represent the ultimate route to evidence-based policy. Finally, as nations transition to Western lifestyles, a dual burden of under- and overnutrition is likely to occur, often with shared underlying factors.

## Recommendations

The portfolio of research to improve nutrition in human beings should be considered from the viewpoint of the potential and the limitations of human and animal study models. The following are recommendations for further research in this field.

1. Epidemiological research to determine to what extent immune competence and susceptibility to infection lags behind physical growth during and following nutritional rehabilitation of malnourished children
  - It is important to establish the relative contributions of overt infection, subclinical infection, inflammation, and host microbial ecology to the genesis and maintenance of stunting and wasting.
  - Nutritional interventions need to be assessed not only in terms of anthropometric responses, but also in terms of effect on body composition, susceptibility to infections, and the ability of children to be resilient to infections.
  - Both basic and clinical research is needed to improve knowledge of protein, energy, and micronutrient requirements during and after infection.
2. Longitudinal birth cohort studies to comprehensively define host physiologic, metabolic, and immunologic phenotypes and microbiota development/maturation in consistently healthy infants living in LMICs where undernutrition/stunting is prevalent
  - The definition of consistently “healthy” is a key initial challenge to address.
  - These studies should ideally encompass the first three years of life, include twins, and frequently sample the microbiota of infants/children, their mothers, fathers, and other co-habitating family members, as well as other sources of microbes (animals, water) to better understand the environmental factors that affect microbiota assembly and maturation.
  - Sampling during diarrheal illnesses and as a function of vaccination is important in these healthy cohorts.
  - These studies will serve as a reference for determining how to better define “healthy growth” in a given population, and for identifying shared as well as distinctive features of healthy growth and microbiota maturation across different cultures/geographic regions. They should yield informative host metabolic and immune as well as microbial biomarkers of healthy growth that can be used to study the pathogenesis of undernutrition, better classify malnourished states, and better define the efficacy of various treatment regimens.
3. Study cohorts of healthy women representing the same populations as those targeted in (2) to obtain new and robust host and microbial biomarkers of maternal nutritional status, define changes in microbial community structure and expressed functions during pregnancy and after delivery, and coincidently determine the biochemical (and microbial) content of breast milk as a function of time after parturition
  - The resulting datasets will serve as references for better understanding of maternal factors that contribute to healthy fetal and postnatal growth, as well as to stunting and other forms of undernutrition.
  - Well-annotated databases need to be established to house study datasets, including clinical metadata.
  - Algorithms, statistical approaches, and visualization tools for analyses of data produced by these time-series studies, within and across different data types, need to be further developed, as do approaches for determining how to adequately power such studies.^108^
  - Procedures used for sample collection, processing, and storage should be deposited in publically accessible locations to facilitate future meta-analyses.
  - Ethical, cultural, and social issues related to consent for future use of samples, plus development of global access policies, need to be addressed in a proactive manner.
4. *Comprehensive longitudinal studies of the effects of a given type of intervention or interventions applied to a particular population studied above*.
  - Study objectives may be treatment and/or prevention of undernutrition (stunting and wasting) in a highly susceptible population. The interventions may involve widely distributed therapeutic foods, locally produced nutrient supplements/foods, or existing or new pre-, pro-, or synbiotics. Whatever the intervention, full characterization and reproducible manufacture of the therapeutic agents used, whether food or microbial, is critical for interpretation and reproduction of the study, as well as for future meta-analyses.
  - An important consideration is the applicability of insights gleaned from studies of SAM, which represents an extreme phenotype or phenotypes, to less severe forms of undernutrition.
  - Extending time-series studies far into the postintervention period allows a critical question to be addressed: how durable are the responses to a given intervention?
  - Biomarkers developed from studies of healthy infants can be applied to address the impact of undernutrition on systems physiology, metabolism, immune function, and microbiota structure/function/maturation during and after an intervention.
  - An important need is to identify ways for assessing mucosal barrier function/gut permeability in humans beyond existing methods.
  - Comparable comments can be made about the interrelationships between the microbiota and undernutrition. Are there common features of microbiota maturation across populations or is there considerable variation? The answer may have important diagnostic and therapeutic implications.
  - As noted in (1) above, it is important to more precisely determine the extent to which immune competence and susceptibility to infection lag behind physical growth during and following nutritional rehabilitation.
  - Clinical trials focusing on preconception, pregnancy, and infancy represent an important route for evidence-based policy on interventions. When designing the latter two types of trials, timing is a key parameter to consider in the context of the mother-infant dyad: for example, should the intervention commence preterm and, if so, when; how long should it be continued during the postnatal period for the mother; when should it be initiated in the infant?
5. Examination of the effects of undernutrition on the epigenome
  - At the moment, there are limited data available on the epigenetic status of individuals with different nutritional or toxin exposure histories.
  - The developmental window in which epigenetic changes occur as a consequence of these histories is also largely unknown; a better understanding of developmental plasticity in this regard may help better define vulnerable populations as well as optimal times for intervention.
  - It is likely that the epigenetic effects of undernutrition will vary geographically, both as a consequence of varying conditions in the environment and the genetic and epigenetic history of the affected populations. Thus, it will be important to compare epigenetic changes in different populations, and to acquire more detailed and complete data linking toxin exposure and nutrition in the same groups of people.
  - Twins discordant for undernutrition and twins concordant for healthy status offer opportunities for characterizing epigenetic effects (as noted above, they can also help illuminate relationships between the microbiota and immune responses, as well as the effects of undernutrition on neurodevelopment/brain metabolism).
6. Development of new noninvasive biomarkers for identifying and classifying EE
  - Currently, no definitive diagnostic tests for EE exist, and assessing therapeutic interventions for EE remains a challenge. The development of noninvasive assays for the diagnosis and classification of EE will help propel research in this field forward.
  - This effort represents the starting point for correlating EE with growth phenotypes, microbiota configuration/maturation, nutrient processing capability, gut barrier function, and other facets of immunity, including vaccine responses.
7. Representative preclinical models
  - Animal models are needed for detailed studies of the effects of maternal nutritional deficiency on intrauterine growth and postnatal development, as are models to improve knowledge of protein, energy, and micronutrient requirements during and after infection (see recommendation 1 above).
  - Models are needed to further elucidate the mechanisms by which micronutrients alter systems physiology and resistance or susceptibility to infection. Common and discrete intracellular pathways involved in the functional activity of single and various combinations of micronutrients need to be identified. Because no single nutrient can solve the problem of micronutrient deficiency in humans, it will be necessary to examine the biological effects of combined micronutrients at various doses.
  - Models are needed to further characterize the relationship between the microbiota (and microbiota maturation) and vaccine efficacy. These studies would be further enhanced if a model for EE is developed (see recommendation 5).
  - Models are needed to further examine the effects of micro- and macronutrient deficiencies on neurodevelopment (e.g., synaptogenesis/synaptic pruning) and brain metabolism.
  - Gnotobiotic animals (mice) harboring transplanted gut microbiota of human donors representative of a targeted human population in an area where undernutrition is very prevalent provide one approach for characterizing the effects of undernutrition of systems physiology, immunity (including vaccine responses), and metabolism. These humanized gnotobiotic animals colonized with microbiota from healthy donors, as well as undernourished individuals whose chronologic age is the same as healthy individuals from the population, can be fed diets representative of that consumed by the population, or representative diets plus nutrient supplements that have been or are going to be given to that population, or that are new candidates for treatment or prevention. Additionally, these mice can be given nutrient supplements plus probiotic consortia. As such, these mice can anticipate or recapitulate the very clinical interventions that are to be or have been applied to the population of interest. The ability to follow these animals under highly controlled conditions should facilitate mechanistic studies, biomarker discovery, lead therapeutic discovery, and translation.
